# Molluscicidal activity and mechanism of toxicity of a novel salicylanilide ester derivative against *Biomphalaria* species

**DOI:** 10.1186/s13071-017-2313-3

**Published:** 2017-08-10

**Authors:** Ping He, Weisi Wang, Benjamin Sanogo, Xin Zeng, Xi Sun, Zhiyue Lv, Dongjuan Yuan, Liping Duan, Zhongdao Wu

**Affiliations:** 1 Department of Parasitology, Zhongshan School of Medicine, Sun Yat-sen University, Guangzhou, 510080 China; 2 Key Laboratory of Tropical Disease Control, Ministry of Education, Guangzhou, 510080 China; 3 Department of Pathogenic Biology and Immunology, Medical School, Xizang Minzu University, Xianyang, 712082 China; 4 National Institute of Parasitic Diseases, Chinese Center for Disease Control and Prevention, WHO Collaborating Centre for Malaria, Schistosomiasis, and Filariasis, Key Laboratory of Parasitology and Vector Biology of the Chinese Ministry of Health, Shanghai, 200025 China; 5 Institute of Microbiology Chinese Academy of Sciences, Beijing, 100101 China

**Keywords:** *Biomphalaria*, *Schistosoma mansoni*, Cercaria, Niclosamide, Salicylanilidate

## Abstract

**Background:**

Schistosomiasis mansoni is one of the most important, but often neglected, tropical diseases transmitted by snails of the genus *Biomphalaria*. Control of the intermediate host snail plays a crucial role in preventing the spread of schistosomiasis. However, there is only one molluscicide, niclosamide, recommended by the World Health Organization. Niclosamide has been used for several decades but is toxic to non-target organisms. Therefore, it is necessary to optimize the scaffold of niclosamide and develop novel molluscicides with enhanced potency and decreased toxicity to non-target organisms.

**Methods:**

In this study, a candidate compound was analyzed by nuclear magnetic resonance and mass spectrometry. The molluscicidal potential against *Biomphalaria* species and cercaricidal potential against *S. mansoni* were evaluated using the immersion method. Furthermore, the preliminary mechanism was studied through cellular enzyme tests and electron microscopy.

**Results:**

5-chloro-2-[(2-chloro-4-nitrophenyl)carbamoyl]phenyl-4-methoxybenzoate (salicylanilidate), a novel salicylanilide ester derivative, was derived from niclosamide. The 50% lethal concentration to *B. glabrata*, *B. straminea* and *B. pfeifferi* was 0.261 mg/l, 0.172 mg/l and 0.241 mg/l, respectively. The effective dose required to completely kill *S. mansoni* cercariae was 0.625 mg/l for salicylanilidate and 0.125 mg/l for niclosamide. However, salicylanilidate was approximately 100-fold less toxic to the fish *Danio rerio* than niclosamide. Furthermore, salicylanilidate reduced the enzymatic activities of nitric oxide synthase (NOS), lactate dehydrogenase (LDH) and acetylcholinesterase (AChE) in the snail, demonstrating that it could affect neurohypophysis transmission and energy metabolism. Severe swelling in the tentacle and deformation of cilia in the tentacle and mantle were observed through scanning electron microscopy. The results of transmission electron microscopy showed that salicylanilidate could damage critical organelles in hepatopancreas tissues, including degeneration of the endoplasmic reticulum and vacuolization in mitochondria. In addition, transcriptional levels of superoxide dismutase (SOD), acid phosphatase (ACP) and NOS in the hepatopancreas were significantly downregulated as shown by real-time quantitative polymerase chain reaction (RT-PCR). These results indicated that the hepatopancreas is a primary target organ of salicylanilidate.

**Conclusions:**

Salicylanilidate not only had deleterious effects on *Biomphalaria* species and *S. mansoni* cercariae but also showed very low toxicity to *D. rerio*, suggesting that it has broad potential applications.

**Electronic supplementary material:**

The online version of this article (doi:10.1186/s13071-017-2313-3) contains supplementary material, which is available to authorized users.

## Background

Schistosomiasis is the second most widespread tropical parasitic disease after malaria. It is endemic in 74 developing countries, with 600 million people at risk of infection [1] and causing approximately 280,000 deaths annually [2]. Schistosomes are the causative agents of schistosomiasis, and *Schistosoma mansoni* is widespread in Africa, South America, the Caribbean and the Middle East [3]. Its life-cycle requires aquatic snails of the genus *Biomphalaria* as obligate intermediate hosts. *Biomphalaria straminea*, an important intermediate host of *S. mansoni* [4], is considered a major invasive species involved in the transmission of schistosomiasis in some invaded habitats, including Brazil, Paraguay, Argentina, Uruguay and the Caribbean [5, 6]. In addition to the Neotropics, *B. straminea* was found in Hong Kong in 1974 [7], and recently, this species has been widely reported in South China [8, 9]. Considering the increases in immigration and tourism, the transmission risk of *S. mansoni* is increasing in these areas [10]. Therefore, it is necessary to monitor *B. straminea* for close surveillance and control.

The application of chemical molluscicide is an efficient strategy for snail control. Multiple synthetic drugs have been developed and used successfully in snail control. However, their development on a large scale is hampered by their environmental effects, toxicity to non-target organisms and high costs for people in most endemic countries [11]. At present, niclosamide is the only molluscicide recommended by the World Health Organization (WHO) since 1960s because of its low toxicity to mammals and low concerns of pesticide residue. However, niclosamide is not suitable for aquaculture due to its toxicity to fish and other aquatic animals [12, 13]. Currently, most modifications of niclosamide have focused on improvement of its solubility by altering formulations or by combining it with surfactants, but little effort has been made to reduce its toxicity through structural modification [14–18]. Therefore, we were prompted to search for efficient, cheap and environmentally friendly compounds with molluscicidal properties.

Our previous studies demonstrated that a salicylanilide ester derivative exhibited similar activity against *Oncomelania hupensis* and negligible toxicity to human kidney cells and *Danio rerio* compared with that of niclosamide [19]. Based on these findings, salicylanilidate was optimized as a novel compound from niclosamide by 4-methoxyphenyl ester substituent at the hydroxyl group and then evaluated for cercaricidal potential against *S. mansoni* and molluscicidal potential against crucial intermediate hosts of *S. mansoni* in the present study. Furthermore, for elucidation of the possible mechanism of toxicity, the in vitro effects of salicylanilidate on various enzyme activities, including acetylcholinesterase (AChE), nitric oxide synthase (NOS), lactate dehydrogenase (LDH), acid phosphatase (ACP) and alkaline phosphatase (AKP), in the soft tissue of *Biomphalaria* snails were examined, and observations of ultrastructure alterations were made using electron microscopy.

## Methods

### Chemistry

Reagents and solvents were purchased from Sigma-Aldrich and were used without further purification. Niclosamide was purchased from Changzhou Yabang-QH Pharmachem Co., Ltd. (Changzhou, China). Melting points were determined with a B-540 Büchi apparatus and remained uncorrected. ^1^H nuclear magnetic resonance (NMR) spectra were recorded on a Bruker AM-400 spectrometer (400 MHz). Chemical shifts (*δ*) were given in ppm relative to tetramethylsilane as an internal standard, and signals were denoted with the following abbreviations: s, singlet; d, doublet; t, triplet; m, multiplet. Mass spectra (MS, ESI) were recorded on a ThermoQ Exactive Orbitrap LC-MS instrument.

### Snails

Three species of the genus *Biomphalaria* were used in this study. *Biomphalaria straminea* (Dunker, 1848) was obtained from East Lake Park of Shenzhen City (22°55′46.51″N, 114°14′90.52″E) and *B. glabrata* (Say, 1818) was provided by the Jiangsu Provincial Institute of Schistosomiasis Prevention and Control, P. R. China. Both were reared in our laboratory from August 2014. In addition, *B. pfeifferi* (Krauss, 1848) was collected from Bamako City (12°65′56″N, 8°3′7″W) in the Republic of Mali in September 2015. All adult snails were maintained at 25 ± 1 °C in plastic tanks (24 × 16 × 9 cm) containing dechlorinated water and fed commercial golden fish food. The dechlorinated water in tanks was replaced twice a week.

### Molluscicidal activity assay

The immersion method was carried out according to WHO procedures [20]. Niclosamide and salicylanilidate were dissolved in a small amount of dimethyl sulfoxide (DMSO) to prepare initial stock solutions at 2 mg/ml and 10 mg/ml, respectively. Preliminary assays were performed in duplicate to determine the effective molluscicidal concentrations. Bioassays were performed via the immersion of laboratory-reared snails in a mixed aqueous solution of the investigated compounds at final concentrations ranging from 0.02 mg/l to 0.15 mg/l for niclosamide and 0.05 mg/l to 0.50 mg/l for salicylanilidate. The final concentrations of DMSO in the working solutions were less than 0.01%. During the test process, all snails were starved under normal diurnal lighting at 25 ± 1 °C. Ten individuals were placed into 800 ml of water for each concentration group in plastic tanks (20 × 13 × 7 cm). The bioassays were performed in duplicate. After a 24-h exposure, the snails were rinsed three times with dechlorinated water and then reared in dechlorinated water without compounds for another 48 h as a recovery period to assess mortality. Niclosamide was used as positive control, and dechlorinated water mixed with DMSO (no compounds) was used as a negative control. Snails were considered dead according to one or more of the following criteria: discoloration, contraction of the hemolymph [21], absence of muscle contraction and heart activity [22], hemorrhage and deterioration of the body tissues [19]. The 50%/90% lethal concentration (LC_50_/LC_90_) values, as well as their 95% confidence limits, were determined through probit analysis.

In addition, molluscicidal activity assays on wild snails was also carried out. As mentioned above, *B. straminea* has spread to Shenzhen, Dongguan and Huizhou from Hong Kong. Therefore, molluscicidal activity was determined against wild snails collected from Shenzhen and Dongguan between October and December in 2016. According to the results of the first analysis, salicylanilidate had nearly 100% molluscicidal activity on laboratory-reared *B. straminea* at 0.35 mg/l; thus, this concentration was tested on all wild snails. In brief, groups of ten wild snails were exposed to salicylanilidate for 24 h at 25 ± 1 °C. One group was exposed to dechlorinated water as a negative control. This test was performed in triplicate. The mortality was assessed after a 48 h recovery period.

### Bioassay of cercaricidal activity

*Schistosoma mansoni* was maintained by passage through male BALB/c mice and *B. glabrata* snails in the laboratory. This bioassay was modified according to a previous reference [23]. In detail, the release of cercaria was performed by exposing positive snails to light for 1 h at 26 ± 2 °C. Freshly shed cercariae were concentrated in 50-ml glass beakers, and their initial density was determined by determining the abundance in 1 ml of sample in triplicate. Then, 10 ml of dechlorinated water containing approximately 500 cercariae was transferred into a 6-well cell culture plate. An appropriate volume of stock solution was added to the cercariae suspension to obtain final concentrations ranging from 0.031 to 0.500 mg/l for niclosamide and 0.156 to 5.000 mg/l for salicylanilidate. Pure dechlorinated water mixed with cercariae was used as a negative control. The lethality to cercaria was observed after a 60-min exposure and divided into four grades according to [23]: complete lethality, strong toxicity, medium toxicity and inactive. The bioassays were performed in duplicate.

### Acute lethal fish toxicity test

The acute lethal fish toxicity test was carried out according to the literature [19]. *Danio rerio* was used as a test animal in the present study. In brief, toxicity tests were performed using apparently healthy fish of equal size and age. Ten fish were apportioned into each group and confined for an exposure interval of 96 h. The vitality of the fish was monitored in real time. Niclosamide was used as a positive control. Negative-control fish were maintained in fresh water. The test was performed in duplicate.

### Cellular enzyme activity and RT-PCR assay

Based on the results of molluscicidal activity tests, all snails were exposed to niclosamide or salicylanilidate at LC_50_ concentrations for 24 h; thereafter, the surviving snails were randomly divided into two groups at the end of each exposure period. Dechlorinated water mixed with DMSO was used as a negative control. The first group was used for cellular enzyme activity detection. Snail shells were first removed, and the soft tissues were placed in Eppendorf tubes. All soft tissues (head, foot and visceral mass) were homogenized (10% *w*/*v*) in 0.1 M phosphate buffer (pH 7.5). The homogenate was centrifuged at 12,000× *g* for 10 min at 4 °C, and the supernatant was stored at −40 °C for biochemical assays [24]. The total protein concentrations of the samples were detected by enhanced BCA protein assay kit (Beyotime Corporation, Shanghai, China). The experimental procedures for the NOS, LDH, ACP, AKP and AChE activity assays were performed in duplicate according to the technical instructions of the diagnostic reagent kits (Nanjing Jiancheng Bioengineering Institute, Nanjing, China).

Another group of surviving snails was prepared for RNA extraction. Snail shells were first removed, and different parts of soft tissues (cephalopodium and hepatopancreas) were then rapidly separated under a stereomicroscope and conserved using TRIzol at -80 °C. Total RNA was extracted using TRIzol (Invitrogen, New York, USA) according to the manufacturer’s instructions. The concentrations of the RNA samples were detected by a NanoDrop 2000 spectrophotometer. Random-primed reverse transcription was performed using 1.5 μg of total RNA according to the Super Script II kit protocol (Promega, Beijing, China). Real-time PCR was performed for NOS, ACP, AKP, AChE, superoxide dismutase (SOD) and actin with a SYBR Green PCR Master Mix kit (Applied Biosystems Corporation, Kusatsu, Japan) in a Light Cycler 480 detection system (Roche). The primers were synthesized by Invitrogen, and the primer sequences were as follows: for NOS (XM_013238377), 5′-ATT CGC TAC GCT GGC TAC-3′ (forward) and 5′-TTC CAT ATT TGG GCT TCC-3′ (reverse); for ACP (XM_013216075), 5′-GTC AAT ATA CCA GTT TTG CC-3′ (forward) and 5′-CGG AAG ACA CTC TCT GAT A-3′ (reverse); for AKP (XM_013212670), 5′-TTA TTA GGT TTG TTT GCA CCC-3′ (forward) and 5′-GTG GCT GTG ATC TAT CCT TCC-3′ (reverse); for AChE (XM_013232876), 5′-TGA CGA CGA CAG GTG ATT-3′ (forward) and 5′-ATG ATG TTG CTC CAG ACC-3′ (reverse); for SOD (EB709552), 5′-TAT CCT TGG CAG ATC ACT-3′ (forward) and 5′-TCA ATT CTT GGT TTC GTC-3′ (reverse); and for actin (U53348), 5′-ACG AGG ACG TAG CCG CTC TTG T-3′ (forward) and 5′-ACC CTG ATG TCT GGG TCT GCC A-3′ (reverse). The fold changes in gene expression were calculated by the comparative Ct method according to reference [25].

### Observation of ultrastructural features and alterations

Based on the results of molluscicidal activity tests, all snails were exposed to niclosamide or salicylanilidate at LC_50_ concentrations for 24 h. Dechlorinated water mixed with DMSO was used as a negative control. At the end of each exposure period, the cephalopodium and hepatopancreas were rapidly separated under a stereomicroscope, washed 3 times with 0.2 M PBS (pH 7.2) and fixed in 0.2 M PBS containing 2.5% glutaraldehyde at 4 °C for 24 h prior to electron microscopy analysis.

For scanning electron microscopy (SEM) [26], specimens were washed 3 times in pH 7.2 PBS and 6 times in cold distilled water to remove glutaraldehyde and dehydrated through an ascending ethanol series (50–100%). Then, ethanol was replaced with acetone and isoamyl acetate, and the specimens were dried using a transitional medium of liquid carbon dioxide. The specimens were coated with platinum using an ion-sputtering apparatus and photographed by a scanning electron microscope (Inspect S, FEI Company, Hillsboro, USA). For transmission electron microscopy (TEM) [27], post-fixation was performed in buffered osmium tetraoxide, followed by dehydration before embedding in resin. Ultrathin sections of the snail hepatopancreas were stained with uranyl acetate and lead citrate. Finally, the hepatopancreas specimens were examined by TEM (Tecnai G^2^ Spirit Twin, FEI Company, Hillsboro, USA).

### Statistical analysis

Standard deviations (SD) were calculated using GraphPad Prism version 5.0 for Windows (GraphPad Software, San Diego, California, USA), and data were expressed as a mean of replicates with SD. Significant differences between the treatment groups used in the bioassays were analyzed by one-way ANOVA (significance at *P* < 0.05) using SPSS19.0 software. The LC_50_ and LC_90_ values were calculated by probit analysis with confidence limits of 95% using SPSS19.0.

## Results

### Chemistry

**Fig. 1:**
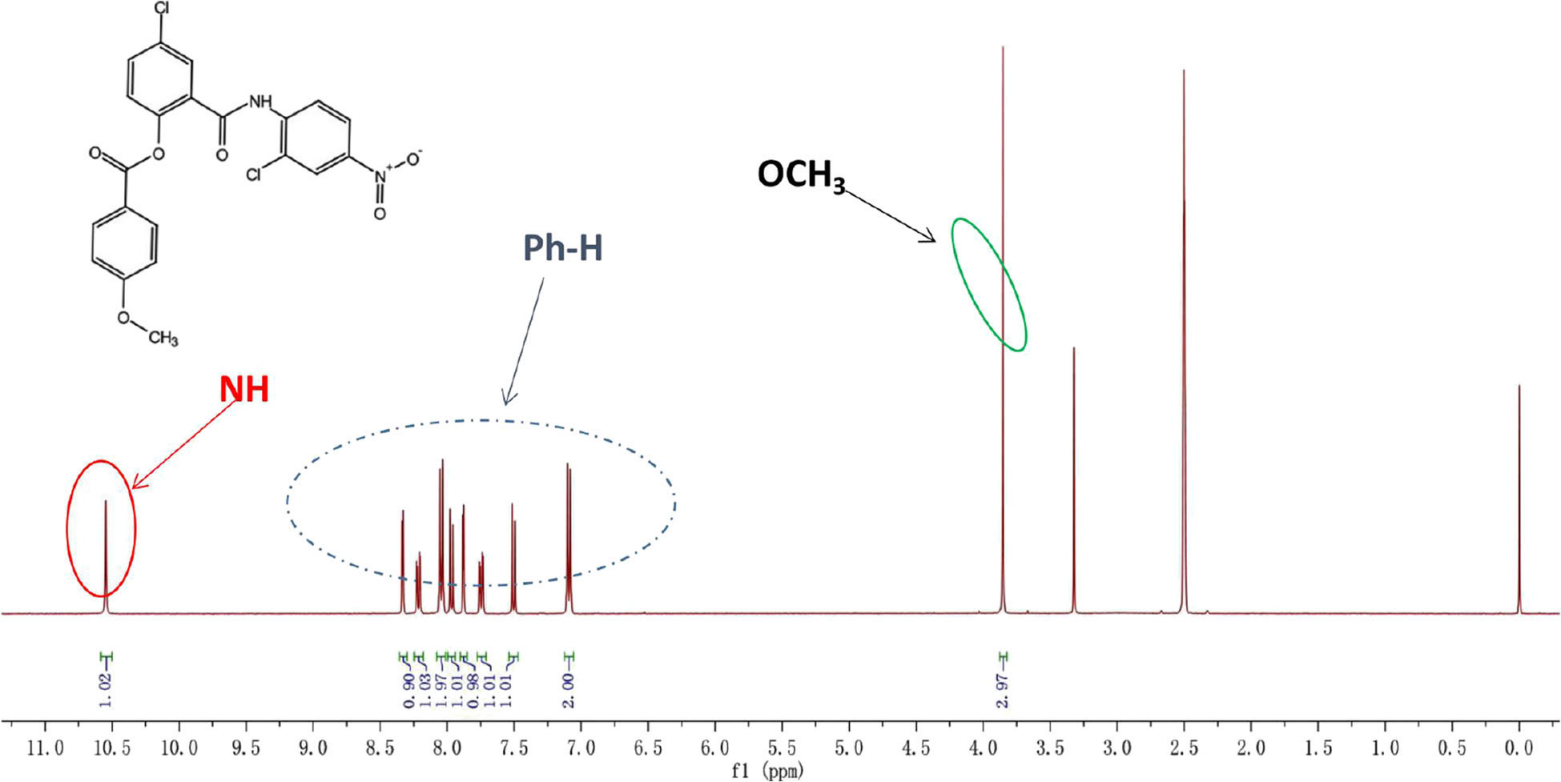
Structure and H^1^NMR analysis of salicylanilidate

Salicylanilidate was synthesized as previously described. White solid, mp: 173.8 to 174.3 °C, LC-MS: m/z 459.009 [M-H^−^] (Additional file 1: Figure S1). As shown in Fig. 1, ^1^H NMR (400 MHz, DMSO) *δ*: 10.55 (s, 1H, NH), 8.34 (d, 1H, Ph-H), 8.20 (dd, 2H, Ph-H), 8.03 (d,1H, Ph-H), 7.96 (dd, 1H, Ph-H), 7.88 (d, 1H, Ph-H), 7.76 (dd, 1H, Ph-H), 7.52 (t, 1H, Ph-H), 7.10 to 7.08 (m, 2H, Ph-H), 3.85 (s, 3H, OCH_3_).

### Molluscicidal activity

To evaluate the molluscicidal potential of salicylanilidate against intermediate hosts of *S. mansoni*, we used three species (*B. glabrata*, *B. straminea* and *B. pfeifferi*) in this study. Interestingly, both salicylanilidate and niclosamide had strong molluscicidal activity against all laboratory-reared snails tested here. After a 24-h exposure in working solution with different concentrations of these compounds, the mortality of the snails was positively correlated with concentration levels. The LC_50_ and LC_90_ values are listed in Table 1. Niclosamide was used as a positive control in this study, and its LC_50_ values for *B. glabrata*, *B. straminea* and *B. pfeifferi* were 0.070 mg/l, 0.049 mg/l and 0.076 mg/l, respectively, which were similar to those in a previous report (0.06 mg/l for both *B. glabrata* and *B. straminea*) [28]. Salicylanilidate displayed an approximately 4-fold-decreased molluscicidal toxicity compared with that of niclosamide. Given the invasion of *B. straminea* in Guangdong Province and the complexity of wild habitats, it is necessary to assess the effect of field application of salicylanilidate. Eight strains of *B. straminea* were collected from Shenzhen and Dongguan and were maintained for one week in the laboratory before the molluscicidal experiment. As shown in Table 2, salicylanilidate exhibited substantial toxicity against wild *B. straminea*, and the lethality was 90–100% at 0.35 mg/l.

**Table 1:** Molluscicidal activity of niclosamide and salicylanilidate against *Biomphalaria* snails by the immersion method (mg/l)

Species	Diameter (mm)	Niclosamide		Salicylanilidate	
		LC_50_	LC_90_	LC_50_	LC_90_
*B. glabrata*	13.87 ± 1.18 (*n* = 110)	0.070 (0.064–0.078)	0.101 (0.089–0.135)	0.261 (0.244–0.278)	0.370 (0.336–0.435)
*B. straminea*	7.97 ± 0.81 (*n* = 108)	0.049 (0.047–0.052)	0.063 (0.060–0.069)	0.172 (0.148–0.201)	0.325 (0.264–0.475)
*B. pfeifferi*	6.47 ± 1.05 (*n* = 66)	0.076 (0.071–0.080)	0.094 (0.087–0.106)	0.241 (0.214–0.268)	0.334 (0.295–0.437)

Values are given as means with 95% confidence intervals for concentration in parentheses

**Table 2:** Molluscicidal effect of salicylanilidate on wild *B. straminea* collected from Guangdong Province

Locality	Coordinates	Number of snails	Diameter (mm)	Mortality (%)
East Lake Park of Shenzhen	22°55′46″N, 114°14′90″E	20^a^	6.33 ± 0.24 (*n* = 20)	100
Hebin Park of Dongguan	22°91′60″N, 114°07′72″E	30	8.40 ± 0.60 (*n* = 30)	100
Shima River of Dongguan	23°03′73″N, 114°10′76″E	30	7.16 ± 0.75 (*n* = 27)	90.00
Tuyang Shenzhen	22°36′55″N, 114°24′14 “E	30	6.39 ± 0.59 (*n* = 30)	100
Dapeng Shenzhen	22°35′49″N, 114°28′21″E	20^a^	7.71 ± 1.03 (*n* = 20)	100
Shuanglong Shenzhen	22°43′48″N, 114°16′23″E	30	8.40 ± 0.74 (*n* = 30)	100
Kuiyong Shenzhen	22°38′06″N, 114°24′35″E	30	6.94 ± 0.58 (*n* = 29)	96.69
Pingshan Shenzhen	22°69′19″N, 114°34′79″E	30	7.58 ± 1.06 (*n* = 29)	96.67

^a^This test was performed in duplicate because of limited samples

### Cercaricidal activity

In addition to molluscicidal activity, the cercaricidal assays demonstrated that salicylanilidate showed strong toxicity to *S. mansoni* cercaria in a dose- and time-dependent manner. After incubation for 60 min, salicylanilidate could completely destroy cercaria at 1.250, 2.500, and 5.000 mg/l, and the cercariae were fractured at the anterior extremity and tail joint. At a concentration of 0.625 mg/l, more than 90% of the cercariae were motionless at the bottom of the dish. Furthermore, salicylanilidate still showed medium toxicity at 0.313 mg/l, but it exhibited weak cercaricidal activity at 0.156 mg/l. For comparison, niclosamide resulted in complete lethality (fractured worms) at 0.250 and 0.500 mg/l, strong toxicity at 0.125 mg/l, and medium toxicity at 0.063 mg/l and was inactive at 0.031 mg/l. Meanwhile, cercariae of the negative control group were active during the whole experiment. Overall, salicylanilidate exhibited substantial insecticidal activity to *S. mansoni* cercariae.

### Acute lethal fish toxicity test

**Fig. 2:**
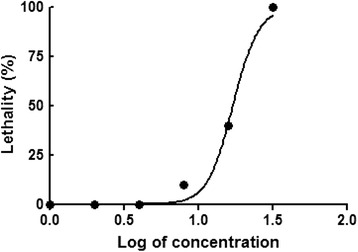
The curvilinear relationship of the logarithm of salicylanilidate concentration and the lethality rate of *D. rerio*

*Danio rerio* is a model organism commonly used in ecotoxicological studies. An acute lethal toxicity assay of *D. rerio* is very useful as an early warning test for monitoring environmentally hazardous chemicals in water. The 96 h LC_50_ value of salicylanilidate was 17.15 mg/l (Fig. 2). According to the Global Harmonization System [29], salicylanilidate is classified as a category 3 toxin to *D. rerio* (10 mg/l < LC_50_ < 100 mg/l), indicating it is substantially less toxic than niclosamide (category 1, LC_50_ of 1 mg/l). These results indicated that the new synthetic salicylanilidate, a molluscicide, is negligibly toxic to *D. rerio*, suggesting it is an environmentally friendly compound with a noticeable improvement over niclosamide.

### Cellular enzyme alterations and RT-PCR

**Fig. 3:**
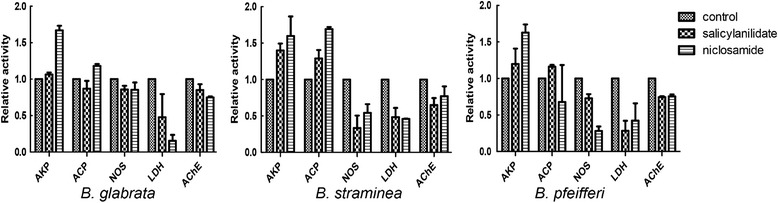
Enzyme-inhibitory activities of salicylanilidate and niclosamide against representative *Biomphalaria* species

**Fig. 4:**
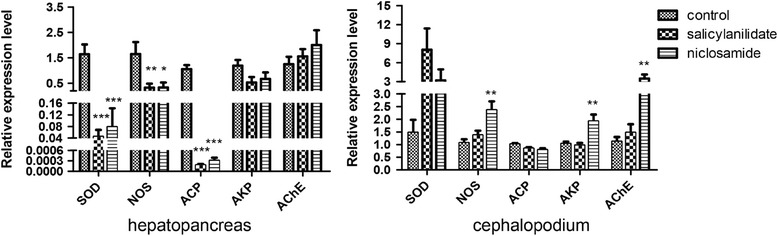
Expression profiles of cellular enzymes of *B. glabrata* exposed to salicylanilidate and niclosamide. Asterisks indicate significant differences between the treatment group and the control group (*P < 0.05, **P < 0.01, ***P < 0.001)

Cellular enzymes are vital to organisms and are often used to evaluate the effects of toxicants on target organisms. The enzymatic activities of AKP, ACP, NOS, LDH and AChE are shown in Fig. 3. Compared with the negative control group, both salicylanilidate and niclosamide could reduce the NOS, LDH and AChE activities and increase the AKP activity after a 24-h incubation at LC_50_ concentrations. Furthermore, expression profiles of these cellular enzymes in *B. glabrata* soft tissues were analyzed (Fig. 4). Compared with the negative control group, the transcriptional levels of ACP, NOS and SOD in the hepatopancreas were significantly downregulated after treatment with the two compounds, and the differences were statistically significant as shown by one-way ANOVA (ACP: *F*_(2, 24)_ = 70.66, *P* < 0.0001; NOS: *F*_(2, 24)_ = 6.916, *P* = 0.0042; and SOD: *F*_(2, 24)_ = 19.86, *P* < 0.0001).

### Observation of ultrastructural features and alterations

**Fig. 5:**
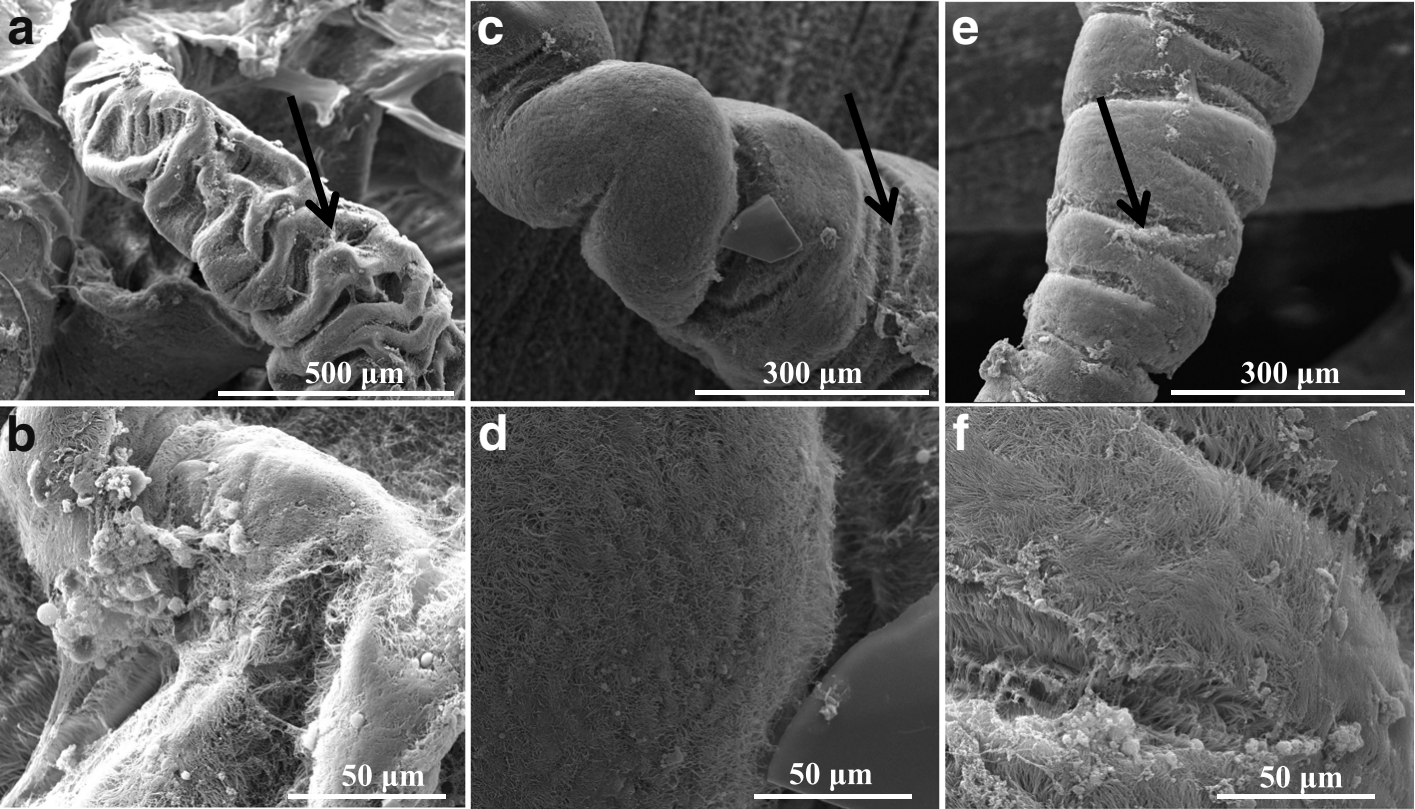
Scanning electron microphotographs of *B. glabrata* tentacle. **a**, **b** The normal ultrastructure of a *B. glabrata* tentacle with plenty of fine cilia and conspicuous surface folds (**a**, *arrow*). **c**, **d** The tentacle folds became flat due to swelling after a 24-h exposure to 0.070 mg/l niclosamide (**c**, *arrow*). The number of cilia decreased significantly, and the cilia were disorderly distributed. **e**, **f** The tentacle folds also became flat after a 24-h exposure to 0.261 mg/l salicylanilidate (**e**, *arrow*), but the cilia density was similar to that of a normal snail

**Fig. 6:**
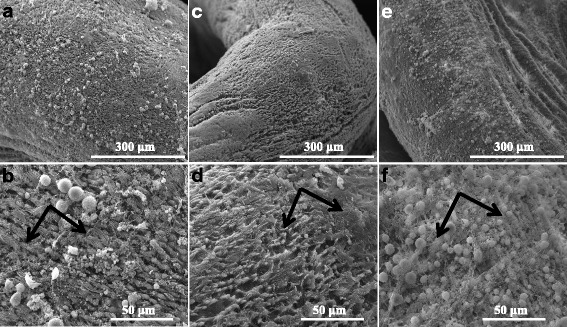
Scanning electron microphotographs of *B. glabrata* mantle. **a**, **b** The normal ultrastructure of *B. glabrata* mantle. There are dense protuberances or spines regularly covered with microvilli on the surface of the mantle (**b**, *arrow*). **c**, **d** Most microvilli were completely destroyed (**d**, *arrow*) and the rough surface was exposed after a 24-h exposure to 0.070 mg/l niclosamide. **e**, **f** The collapsed microvilli of the mantle were distributed irregularly after a 24-h exposure to 0.261 mg/l salicylanilidate (**f**, *arrow*)

**Fig. 7:**
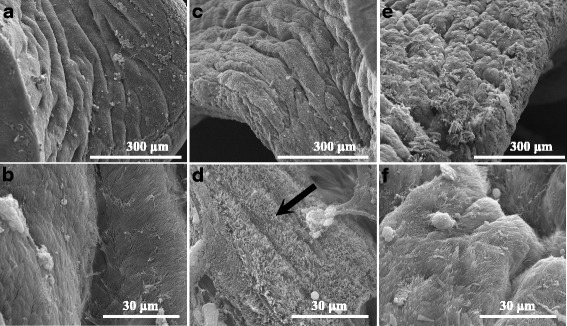
Scanning electron microphotographs of *B. glabrata* foot plantaris. **a**, **b** The normal ultrastructure of *B. glabrata* foot plantaris regularly coated with numerous microvilli and with notable surface folds. **c**, **d** The microvilli became curly and disorderly after a 24-h exposure to 0.070 mg/l niclosamide (**d**, *arrow*). **e**, **f** There was no obvious alteration in salicylanilidate group compared with snails of the control group

**Fig. 8:**
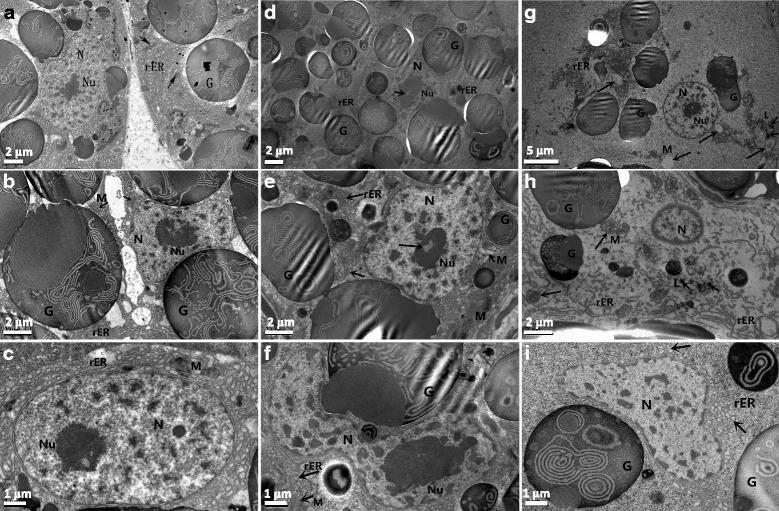
Transmission electron microscope observations of hepatopancreas cell organelles of *B. glabrata.* The magnification times of the photos are as follows: **a** and **d** (×3900; *scale-bars*: 2 μm), **g** (×2850; *scale-bars*: 5 μm), **b**, **e** and **h** (×5800; *scale-bars*: 2 μm), and **c**, **f** and **i** (×7900; *scale-bars*: 1 μm). **a**–**c** The normal ultrastructural features of *B. glabrata* hepatopancreas cells. Abundant rough endoplasmic reticulum (rER) and long oval or irregular nuclei (N) were observed in cells, as well as mitochondria (M) and circular secretion granules (G) in the cytoplasm. Clear nucleolus (Nu) and exiguous heterochromatin were distributed in the nucleus. **d**–**f** The ultrastructural alterations of hepatopancreas cells exposed to niclosamide included clumped heterochromatin in the nucleus (**d**–**f**), focal lysis of the nucleolus (**e**, *large arrow*), and polymorphic nuclei (**f**), as well as vacuolization of the mitochondria (**e**, *small arrow*), partially destroyed endoplasmic reticulum (E/F, *moderate arrow*) and degeneration of secretion granules. **g**–**i** The ultrastructural alterations of hepatopancreas cells exposed to salicylanilidate. The endoplasmic reticulum was seriously damaged or showed obvious vacuolization in all three photos (**g**–**i**). Both the nucleus and mitochondria (*arrow*) were swollen in (**g**), but the nucleolus was still clear. Lysosomes (L) appeared in **g** and **h** (*small arrow*). Other changes included marginalized heterochromatin (**h**), polymorphic nuclei (**i**) and degeneration of secretion granules (**g**)

The ultrastructural features and alterations of the tentacle, mantle, foot plantaris and hepatopancreas of *B. glabrata* are shown in Figs. 5, 6, 7, and 8, respectively. The SEM photographs revealed that both compounds could lead to swelling of the tentacle and deformation of cilia in the mantle, but niclosamide showed increased destruction of the soft tissues compared with salicylanilidate at LC_50_ concentrations. The TEM photographs revealed that salicylanilidate predominantly caused rough endoplasmic reticulum damage, heterochromatin aggregation and vacuolization of the mitochondria, as well as polymorphic changes of the nucleus (Fig. 8h, i). In contrast, niclosamide primarily led to polymorphic alterations of the nucleus, substantial heterochromatin aggregation, and partial damage to the rough endoplasmic reticulum (Fig. 8d, f).

## Discussion

Snail control by molluscicide remains one of the most effective measures of schistosomiasis control. Niclosamide has been recommended by WHO as a molluscicide since the 1960s, but there are some limitations associated with its application, such as toxicity to non-target organisms. To overcome these problems, we derived salicylanilidate as an optimized novel compound from niclosamide by 4-methoxyphenyl ester substitution at the hydroxyl group. In immersion assays, all snails were exposed to salicylanilidate for 24 h, and the LC_50_ values for *B. glabrata*, *B. straminea* and *B. pfeifferi* were 0.261 mg/l, 0.172 mg/l and 0.241 mg/l in the laboratory, respectively. Considering the differences in habitats and sensitivities to molluscicides between wild snails and laboratory-reared snails, 8 strains of wild *B. straminea* collected from Dongguan and Shenzhen were used to evaluate molluscicidal activity. The lethality of salicylanilidate was 90–100% at 0.35 mg/l, exhibiting similar molluscicidal activity to that on laboratory-reared snails. In addition, 0.625 mg/l of salicylanilidate showed the same cercaricidal effect as 0.125 mg/l of niclosamide. Overall, these results demonstrated that salicylanilidate reduced the survival of *B. glabrata*, *B. straminea* and *B. pfeifferi*. Importantly, salicylanilidate displayed an approximately 100-fold-decreased fish toxicity to *D. rerio* compared with that of niclosamide, indicating that salicylanilidate, to some degree, does not have the disadvantage of high fish toxicity of niclosamide and could be used as molluscicide in aquaculture areas.

Cellular enzymes play vital roles in maintaining physiological activities. Recently, several enzymes have been used as biomarkers to assess the effects of toxicants or molluscicides on aquatic animals [24, 30–32]. To elucidate the molecular mechanism associated with the toxicity of salicylanilidate to *Biomphalaria* species, we measured the enzyme activities of ACP, AKP, NOS, LDH and AChE. After a 24-h exposure at LC_50_ concentration, salicylanilidate reduced the enzyme activities of NOS, LDH, and AChE and increased the activity of AKP. The average change in enzymatic activity in the salicylanilidate group was similar to that of niclosamide (Fig. 3). These results were consistent with previous reports showing that niclosamide could inhibit NOS, LDH, and AChE activities in HEK293 cells [19] and *O. hupensis* [33] and a report indicating that aqueous extracts of *Croton tiglium* could significantly alter the activities of LDH, AChE, ACP and AKP in the nervous tissue and hepatopancreas of *Lymnaea acuminata* [34]. NOS can catalyze L-arginine and oxygen to produce the gaseous signaling molecule nitric oxide (NO). NO plays an important role in neurotransmission for snails [35] and major roles in resistance to pathogens and immune regulation in molluscs. LDH is a representative enzyme in the anaerobic glycolytic pathway and plays a crucial role in the final step of glycolysis. Glycolytic enzymes are essential for energy metabolism of the snail. Acetylcholine is a neurotransmitter in the nervous system, and AChE has a critical function in catalyzing the hydrolysis of acetylcholine to maintain the normal conduct of nerve impulses. AChE is the primary target of organophosphate and carbamate pesticides and can be used as a potent biomarker of pesticide toxicity to snails [36, 37]. In this study of molluscicidal activity, the movement speed of all snails was significantly decreased after immersion with salicylanilidate, which may be related to the dysfunctional neurotransmission and lack of energy supply caused by decreased AChE, NOS and LDH. Therefore, physiological dysfunction caused by changes of important enzymes may be involved in the toxicity of salicylanilidate against snails.

In addition to cellular enzymes, ultrastructural features of the hepatopancreas have been widely investigated in molluscicide studies. The hepatopancreas is a pivotal detoxification organ for snail and is particularly vulnerable to injuries. The ultrastructure of the hepatopancreas was noticeably altered by salicylanilidate, including degeneration or destruction in the endoplasmic reticulum, vacuolization in the mitochondria and heterochromatin aggregation in the nucleus. The endoplasmic reticulum is closely involved in synthesis and secretion processes in the hepatopancreas. Mitochondria, a major site for sugars, amino acids and fatty acid oxidation, play crucial roles in energy supply for biological activities. Interestingly, there was little difference between the molluscicidal mechanisms of the two compounds. Salicylanilidate predominantly led to the destruction of the rough endoplasmic reticulum, and to moderate heterochromatin aggregation (Fig. 8h, i). In contrast, niclosamide primarily led to polymorphic alterations of the nucleus, focal lysis in the nucleolus and increased heterochromatin aggregation, as well as moderate destruction of the endoplasmic reticulum (Fig. 8d, f). *Biomphalaria* snails have no operculum; thus, their cephalopodia were continuously in contact with the molluscicide during the toxicity test. Therefore, gene expression profiles of several cellular enzymes in the cephalopodium and hepatopancreas were further compared using RT-PCR. Transcriptional levels of NOS, ACP and SOD in the hepatopancreas were significantly downregulated compared with those in cephalopodium (Fig. 4). ACP, a lysosomal enzyme, plays critical roles in catabolism, pathological necrosis, autolysis and phagocytosis [38]. SOD can alternatively catalyze the dismutation of the superoxide radical, which has pivotal function in antioxidant defense. Therefore, the hepatopancreas may be a critical target tissue of salicylanilidate.

## Conclusions

Salicylanilidate was synthesized as a novel salicylanilide ester derivative in this study, and it exhibited strong molluscicidal potential against *Biomphalaria* species and cercaricidal potential against *S. mansoni*. The results indicated that the dysfunction of cellular enzyme activities, neurotransmitter conduction and energy metabolism may be involved in the toxicity mechanism of salicylanilidate. Notably, salicylanilidate displayed negligible toxicity to *D. rerio*, which suggested that it could be developed as a promising drug against *S. mansoni* at the transmission stages and would be useful as a candidate molluscicide in aquaculture. Further investigations of the toxicity of salicylanilidate to more species and the effects of field application are in progress.

## Additional file

Additional file 1: Figure S1. The high resolution mass spectra analysis of salicylanilidate. (TIFF 1218 kb)

## Abbreviations

AChE: Acetylcholinesterase
ACP: Acid phosphatase
AKP: Alkaline phosphatase
DMSO: Dimethyl sulfoxide
LC: Liquid chromatography
LC_50_/LC_90_: The 50/90% lethal concentration
LDH: Lactate dehydrogenase
MS: Mass spectra
NMR: Nuclear magnetic resonance
NOS: Nitric oxide synthase
RT-PCR: Real-time quantitative polymerase chain reaction
SD: Standard deviation
SEM: Scanning electron microscopy
SOD: Superoxide dismutase
TEM: Transmission electron microscopy
WHO: World Health Organization
